# Supplementary material for: Lactate dehydrogenase D is a general dehydrogenase for D-2-hydroxyacids and is associated with D-lactic acidosis
Source: Nat Commun. 2023 Oct 20;14:6638. doi: 10.1038/s41467-023-42456-3 (PMC10589216; doi:10.1038/s41467-023-42456-3)
Supplement: Supplementary file 1 — Supplementary Information [file 41467_2023_42456_MOESM1_ESM.pdf]

Supplementary Information

Lactate dehydrogenase D is a general dehydrogenase for D-2-hydroxyacids and is associated with D-lactic acidosis

Shan Jin[1,#], Xingchen Chen[1,#], Jun Yang[1], and Jianping Ding[1,2,*]

[1] State Key Laboratory of Molecular Biology, Shanghai Institute of Biochemistry and Cell Biology, Center for Excellence in Molecular Cell Science, University of Chinese Academy of Sciences, Chinese Academy of Sciences, 320 Yue-Yang Road, Shanghai 200031, China

[2] School of Life Science and Technology, ShanghaiTech University, 393 Middle Huaxia Road, Shanghai 201210, China

[#] These authors contributed equally: Shan Jin, Xingchen Chen

[*] To whom correspondence should be addressed. E-mail: jpding@sibcb.ac.cn

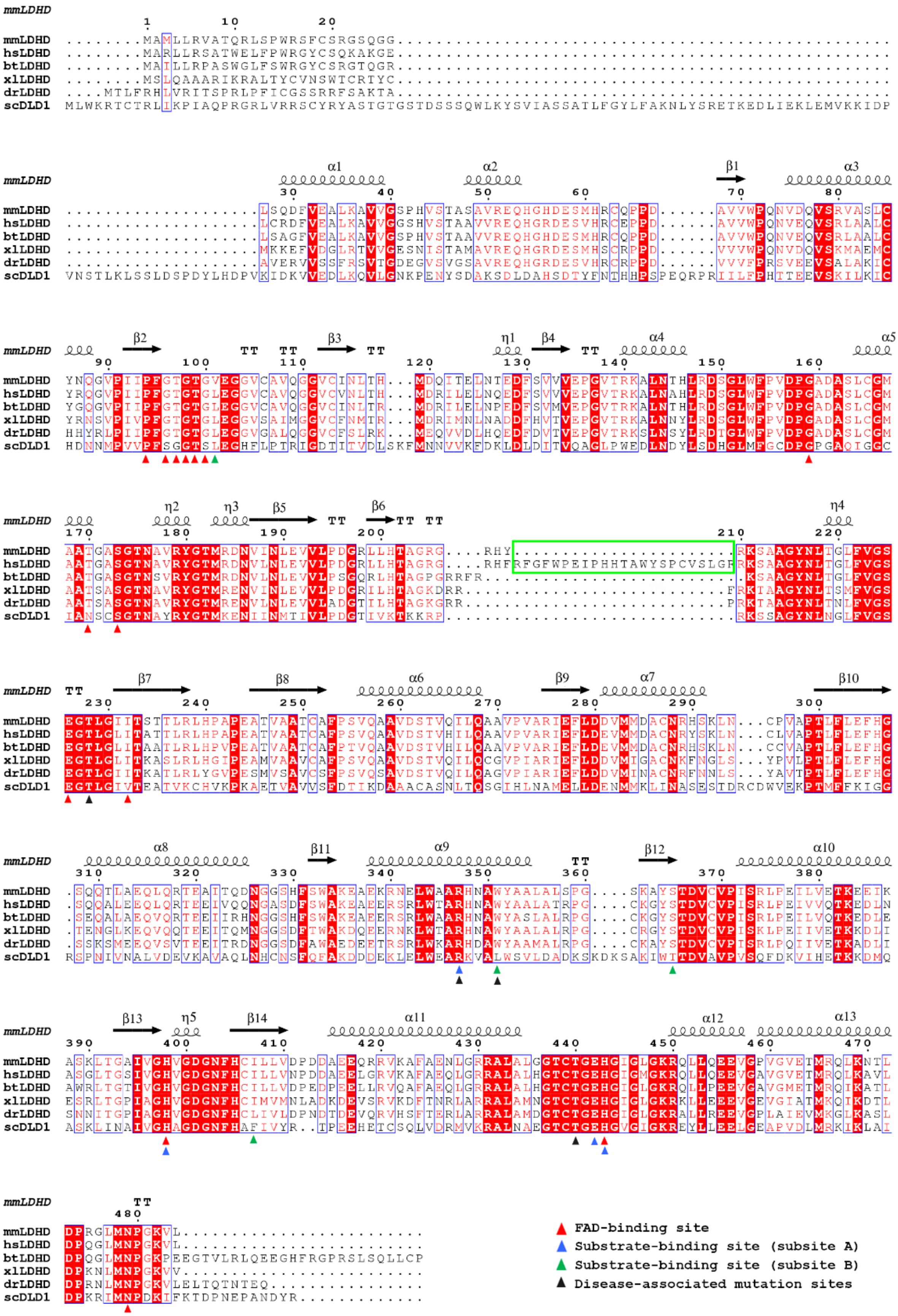
mmLDHD
hsLDHD
btLDHD
xlLDHD
drLDHD
scDLD1
FAD-binding site
Substrate-binding site (subsite A)
Substrate-binding site (subsite B)
Disease-associated mutation sites

Supplementary Fig 1. Sequence alignment of LDHD from six representative eukaryotes. Amino acid sequences of FAD-dependent D-lactate dehydrogenases from *H. sapiens* (hsLDHD, Uniprot: Q86WU2), *M. musculus* (mmLDHD, Uniprot: Q7TNG8), *B. taurus* (btLDHD, Uniprot: Q148K4), *X. laevis* (xlLDHD, Uniprot: A0A1L8GLK1), *D. rerio* (drLDHD, Uniprot: F1QXM5), and *S. cerevisiae* (scDLD1, Uniprot: P32891) are aligned. The 23-residue insertion of hsLDHD is indicated by green box. The secondary structures of mmLDHD are placed on the top of the sequence alignment. Residues of mmLDHD involved in the FAD binding are indicated by red triangles. Residues of mmLDHD composing the substrate-binding subsites A and B are indicated by blue and green triangles, respectively. The disease-associated mutations of human LDHD are indicated by black triangles.

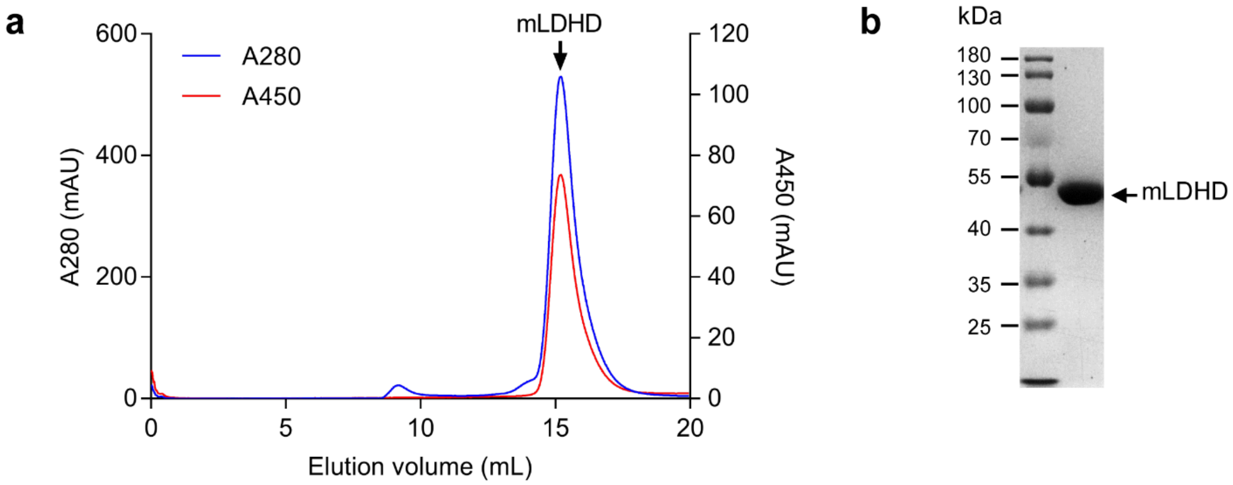

Supplementary Fig 2. Biochemical characterization of mLDHD. a. Size-exclusion chromatography analysis of mLDHD. The absorbances at 280 nm and 450 nm are monitored. **b.** SDS-PAGE analysis of mLDHD. The purified mLDHD protein has high purity and homogeneity and exists as a monomer in solution. Source data are provided as a Source Data file.

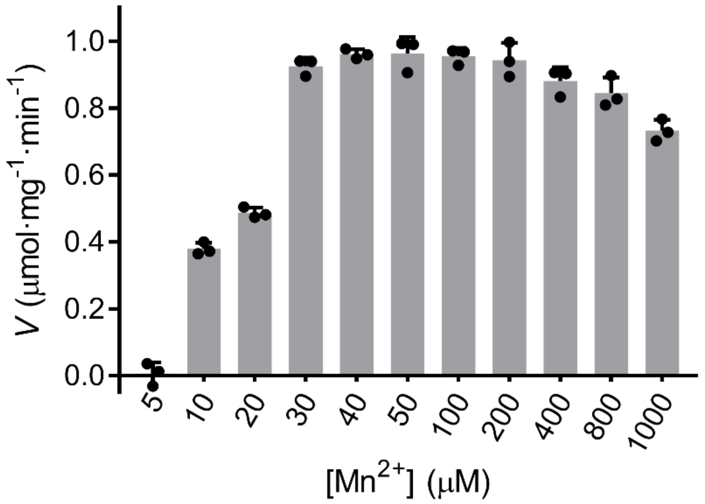

Supplementary Fig 3. Specific activity of mLDHD towards D-LAC with varied Mn^{2+} concentrations at the standard reaction conditions. The error bars show the standard errors of the mean (SEM) of three independent measurements. Source data are provided as a Source Data file.

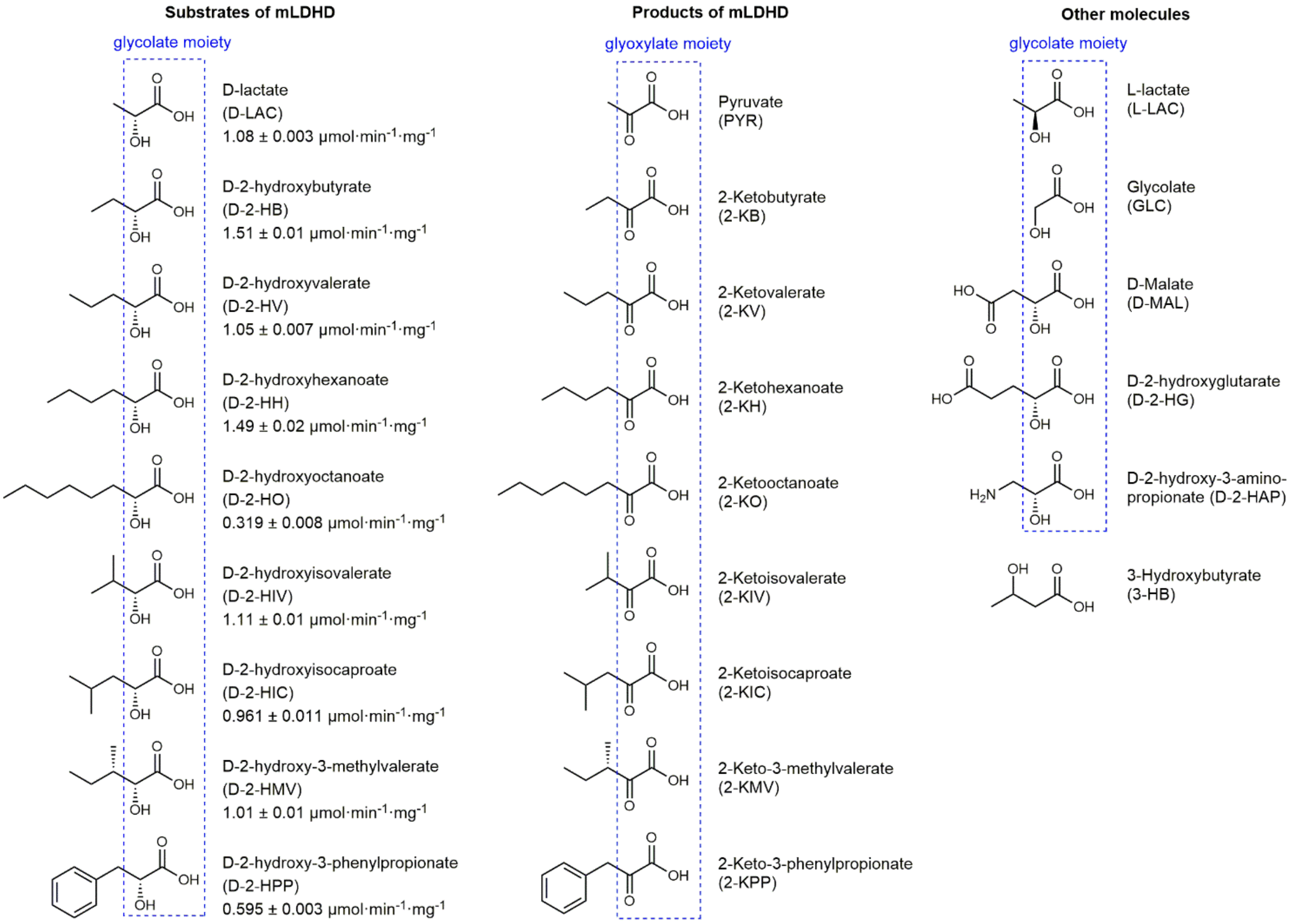

Supplementary Fig 4. Chemical structures of organic acids used in this study. The common glycolate moiety of 2-hydroxyacids and the common glyoxylate moiety of 2-ketoacids are indicated with dashed boxes. The specific activity of mLDHD towards different substrates (1 mM) is listed beside the substrate structure (n = 3 independent experiments).

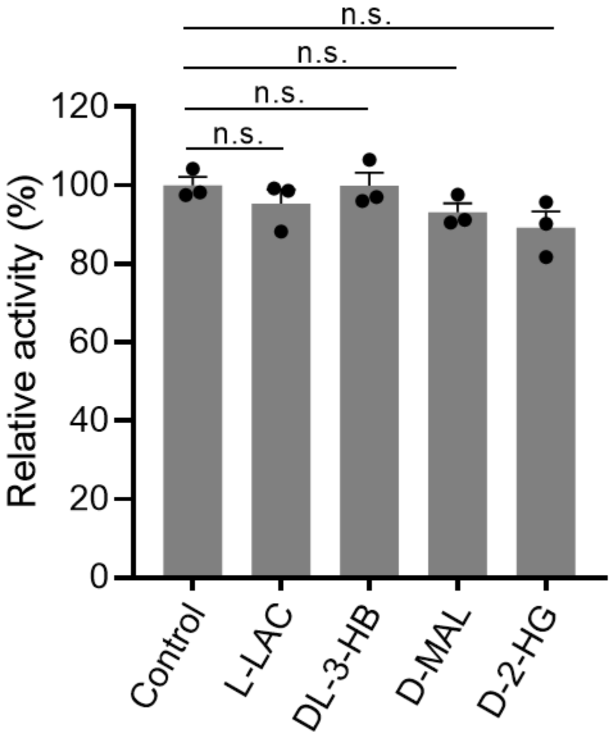

Supplementary Fig 5. Specific activity of mLDHD towards D-LAC in the absence and presence of several substrate analogs. The specific activity of mLDHD towards D-LAC (100 μM) was measured in the absence (control) and presence of indicated substrate analogs (1 mM) including L-lactate (L-LAC), DL-hydroxybutyrate (DL-3-HB), D-malate (D-MAL), and D-2-hydroxyglutarate (D-2-HG). The data are presented as percentage of the specific activity in the presence of substrate analog relative to that in the absence of substrate analog (control). The error bars represent the standard errors of the mean (SEM) of three independent experiments (n = 3). The *p* values were calculated with two-sided Student's *t*-test (0.33 for L-LAC, 0.98 for DL-3-HB, 0.09 for D-MAL and 0.08 for D-2-HG). n.s., not significant ($p > 0.05$). Source data are provided as a Source Data file.

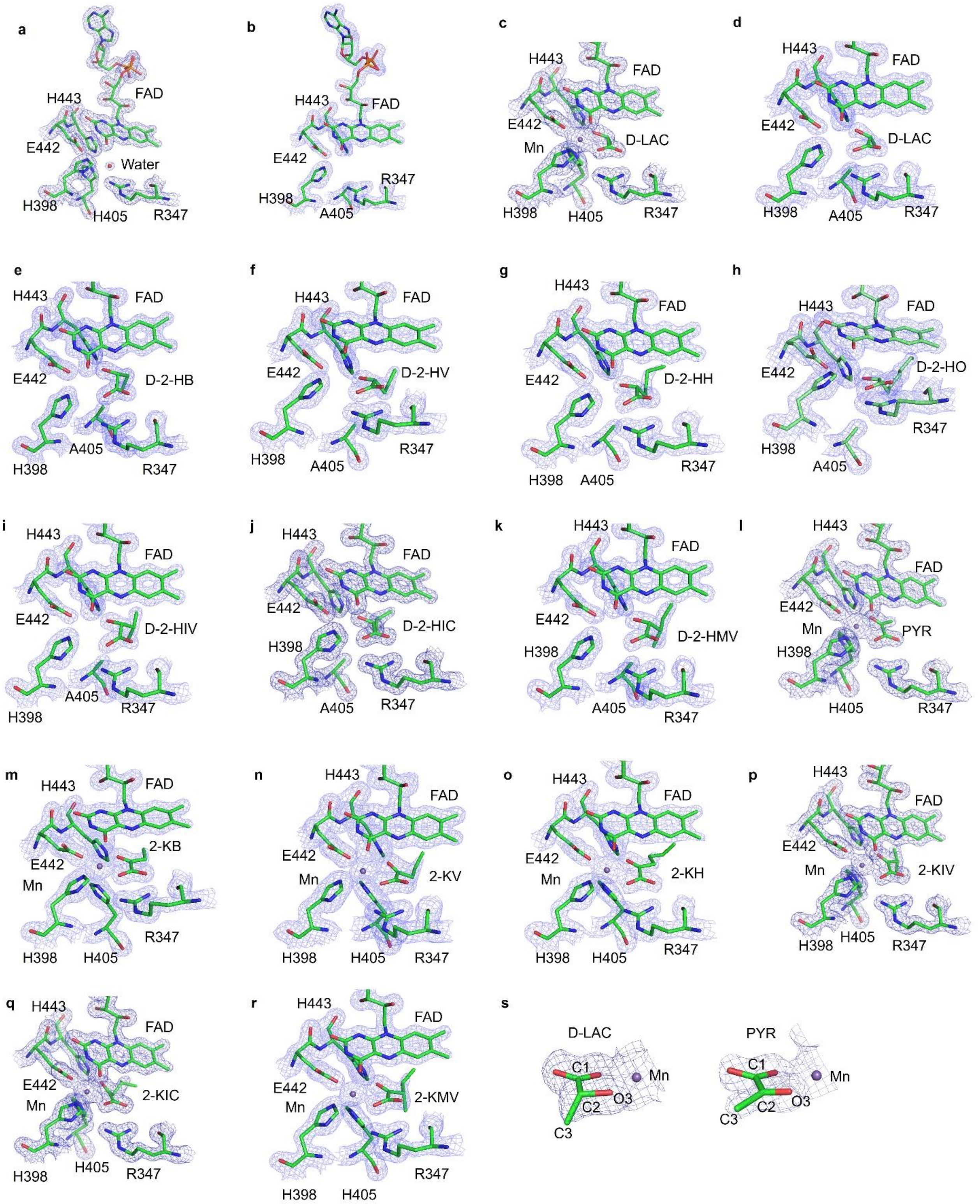

Supplementary Fig 6. Representative simulated annealing composite *2Fo-Fc* omit maps (1.5 σ contour level) for the active sites in different mLDHD structures. The FAD, substrate or product, and key residues at the active site are shown with stick models, and the metal ion and water molecule are shown as cyan sphere and red sphere, respectively. **a.** The structure of the FAD-bound WT mLDHD. **b.** The structure of the FAD-bound $mLDHD_{H405A}$. **c.** The structure of WT mLDHD in complex with FAD, Mn^{2+} and D-lactate (D-LAC). **d.** The structure of $mLDHD_{H405A}$ in complex with

FAD and D-LAC. **e-k.** The structures of mLDHD$_{H405A}$ in complexes with FAD and different substrates: D-2-hydroxybutyrate (D-2-HB, **e**), D-2-hydroxyvalerate (D-2-HV, **f**), D-2-hydroxyhexanoate (D-2-HH, **g**), D-2-hydroxyoctanoate (D-2-HO, **h**), D-2-hydroxyisovalerate (D-2-HIV, **i**), D-2-hydroxyisocaproate (D-2-HIC, **j**), and D-2-hydroxy-3-methylvalerate (D-2-HMV, **k**). **l-r.** The structures of WT mLDHD in complexes with FAD, Mn^{2+} and different products: pyruvate (PYR, **l**), 2-ketobutyrate (2-KB, **m**), 2-ketovalerate (2-KV, **n**), 2-ketohexanoate (2-KH, **o**), 2-ketoisovalerate (2-KIV, **p**), 2-ketoisocaproate (2-KIC, **q**), and 2-keto-3-methylvalerate (2-KMV, **r**). **s.** Comparison of the electron density maps for D-LAC in the mLDHD$^{FAD+Mn+D\text{-}LAC}$ structure and PYR in the mLDHD$^{FAD+Mn+PYR}$ structure showing the co-planarity and non-planarity of the C2 atom with the C1, C3 and O3 atoms, respectively.

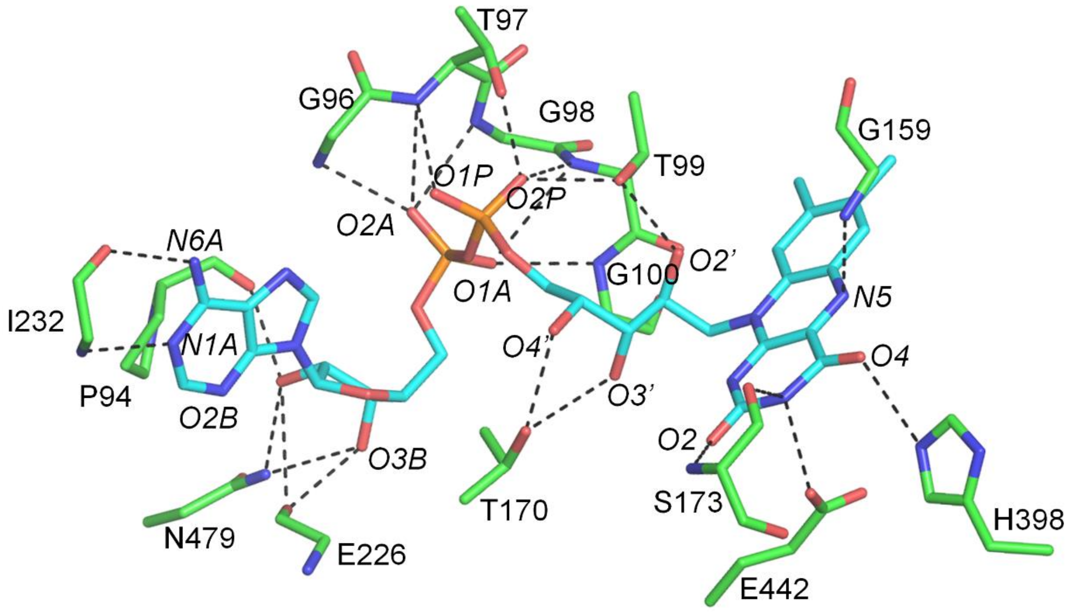

Supplementary Fig 7. Hydrogen-bonding interactions between FAD and the surrounding residues in the FAD-bound WT mLDHD structure. FAD is shown as cyan stick and the protein residues in green stick. The hydrogen bonds are indicated by dashed lines.

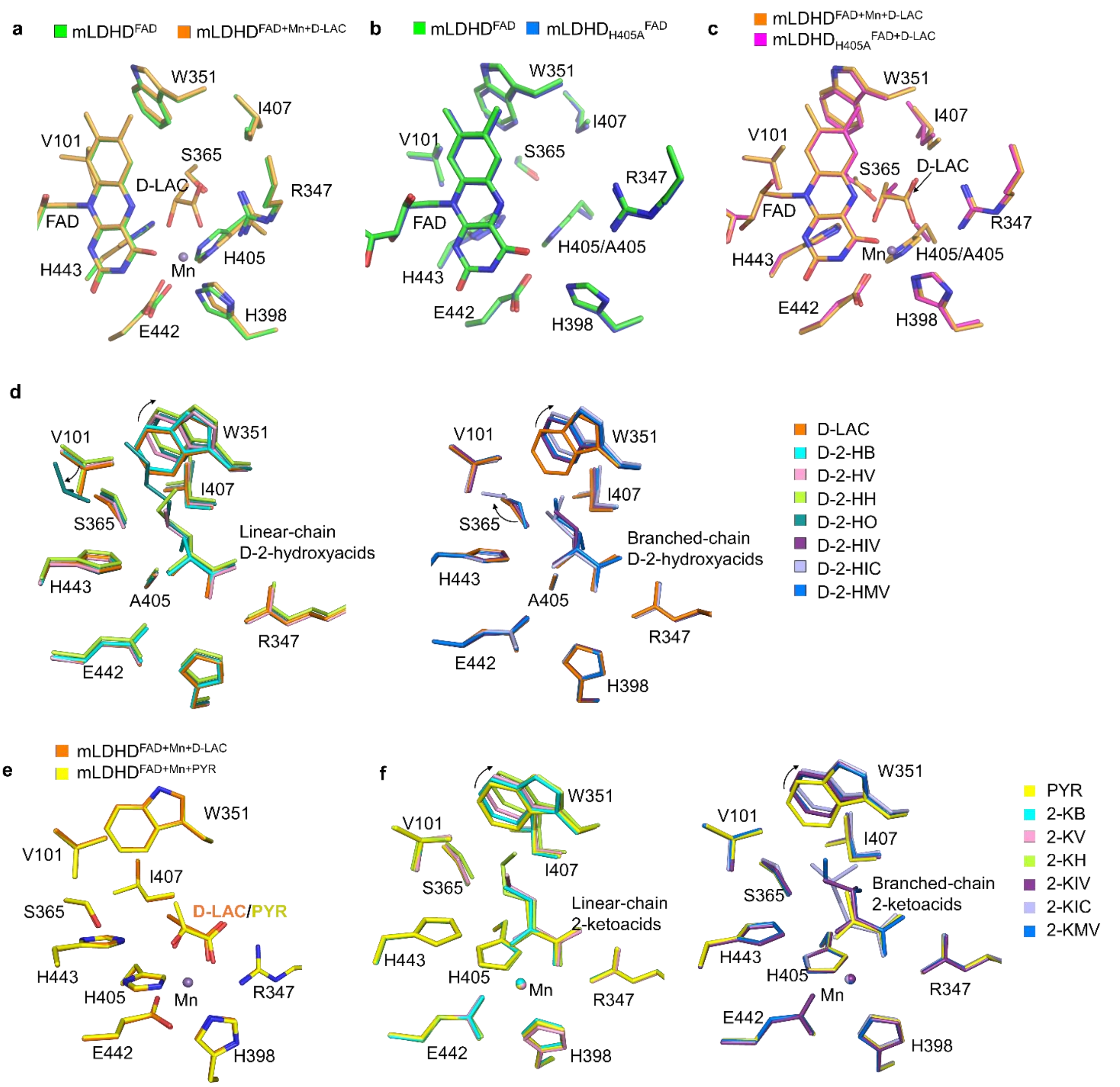

Supplementary Fig 8. **Comparisons of the active sites in different mLDHD structures. a.** Comparison of the active sites in the WT mLDHD^{FAD} and mLDHD$^{FAD+Mn+D\text{-}LAC}$ structures. **b.** Comparison of the active sites in the WT mLDHD^{FAD} and mutant mLDHD$_{H405A}^{FAD}$ structures. **c.** Comparison of the active sites in the WT mLDHD$^{FAD+Mn+D\text{-}LAC}$ and mutant mLDHD$_{H405A}^{FAD+D\text{-}LAC}$ structures. **d.** Comparison of the active sites in different substrate-bound mLDHD$_{H405A}$ structures showing the conformational changes of the key residues forming the substrate-binding subsite B to varied extents along with the increase of the size of the hydrophobic moiety attached to the C2 atom of substrate. Left: comparison of the active sites in structures bound with D-LAC and other D-2-hydroxyacid substrates with linear aliphatic moieties including D-2-hydroxybutyrate (D-2-HB), D-2-hydroxyvalerate (D-2-HV), D-2-hydroxyhexanoate (D-2-HH), and D-2-hydroxyoctanoate (D-2-HO). Right: comparison of the active sites in structures bound with D-LAC and other D-2-hydroxyacid substrates with branched aliphatic moieties including D-2-hydroxyisovalerate (D-2-HIV), D-2-hydroxyisocaproate (D-2-HIC), and D-2-hydroxy-3-methylvalerate (D-2-HMV). **e.** Comparison of

the active sites in the WT $mLDHD^{FAD+Mn+D\text{-}LAC}$ and $mLDHD^{FAD+Mn+PYR}$ structures. **f.** Comparison of the active sites in different product-bound mLDHD structures showing the conformational changes of the key residues forming the substrate-binding subsite B to varied extents along with the increase of the size of the hydrophobic moiety attached to the C2 atom of product. Left: comparison of the active sites in structures bound with PYR and other 2-ketoacid products with linear aliphatic moieties including 2-ketobutyrate (2-KB), 2-ketovalerate (2-KV), and 2-ketohexanoate (2-KH). Right: comparison of the active sites in structures bound with PYR and other 2-ketoacid products with branched aliphatic moieties including 2-ketoisovalerate (2-KIV), 2-ketoisocaproate (2-KIC), and 2-keto-3-methylvalerate (2-KMV).

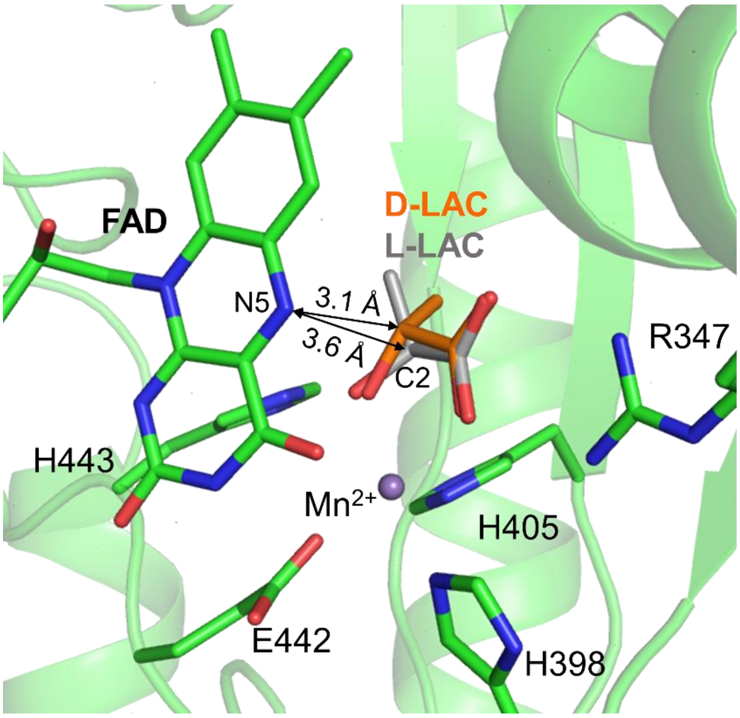

Supplementary Fig 9. **Docking of L-LAC into the active site of the mLDHD$^{FAD+Mn+D\text{-}LAC}$ structure.** The docked L-lactate (L-LAC, grey) and the bound D-lactate (D-LAC, orange) are superimposed.

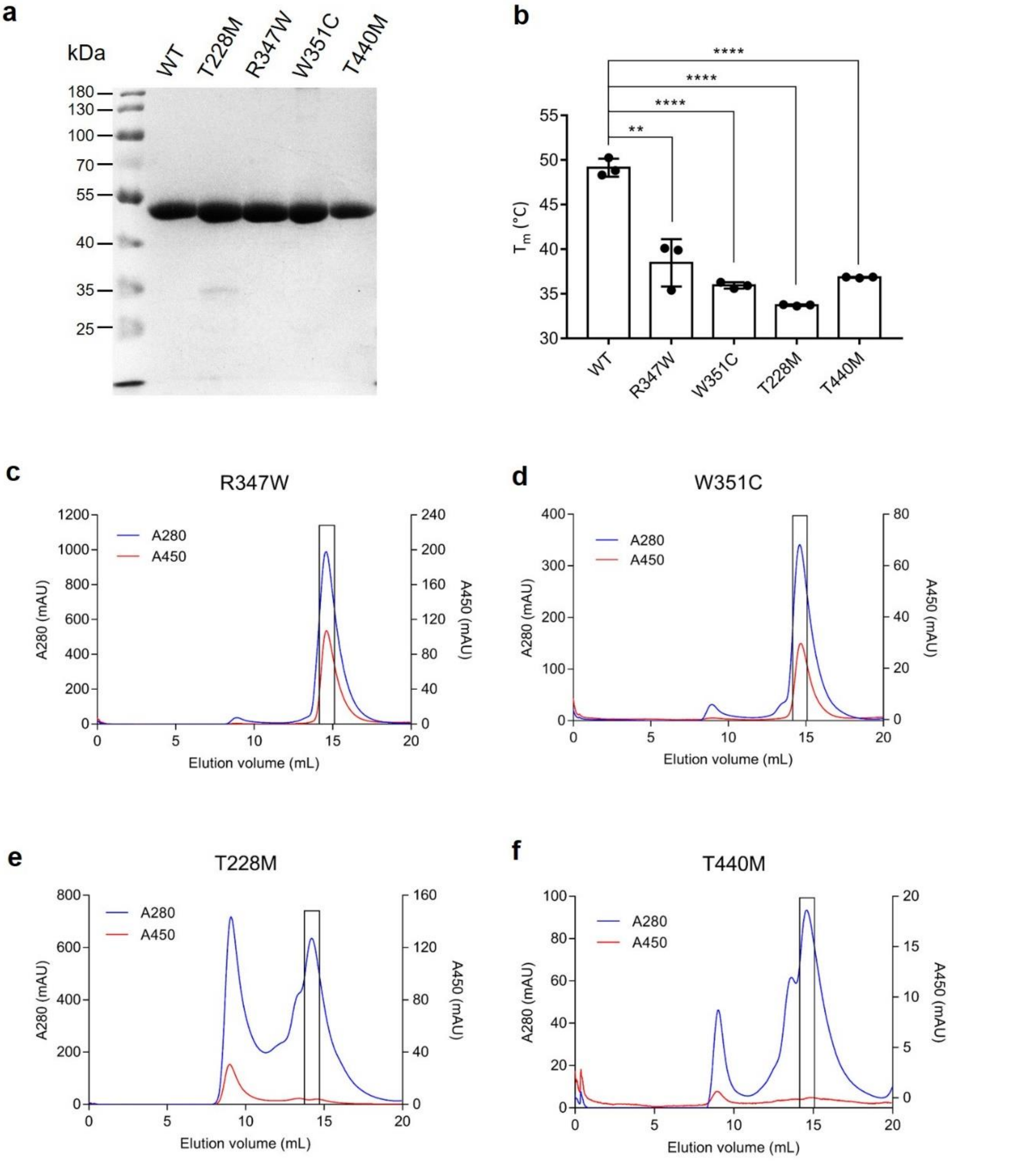

Supplementary Fig 10. Biochemical analysis of the mLDHD mutants containing mutations corresponding to the disease-associated mutations of hLDHD. a. SDS-PAGE analysis of WT mLDHD and the T228M, R347C, W351C, and T440M mLDHD mutants. **b.** Thermostability analysis of WT mLDHD and the T228M, R347C, W351C, and T440M mLDHD mutants (n = 3 independent experiments). The *p* values were calculated with two-sided Student's *t*-test. **, $p<0.01$ (2.9×10^{-3} for R347W); ****, $p<0.0001$ (2.8×10^{-5} for W351C, 1.2×10^{-5} for T228M and 3.0×10^{-5} for T440M). **c-f.** Elution curves of the R347C (**c**), W351C (**d**), T228M (**e**), and T440M (**f**) mLDHD mutants from the size-exclusion chromatography analysis. The absorbances at 280 nm and 450 nm were monitored. The peaks corresponding to the target protein are indicated by boxes. Source data are provided as a Source Data file.

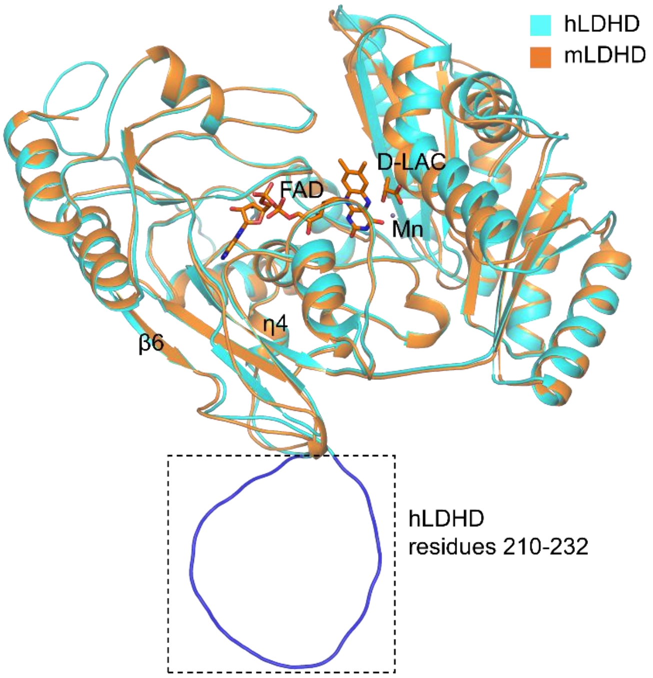

Supplementary Fig 11. Comparison of the Alphafold2 predicted hLDHD structure (Q86WU2) and the mLDHD$^{FAD+Mn+D\text{-}LAC}$ structure. The Alphafold2 predicted hLDHD structure is colored in cyan with the insertion in blue. The mLDHD$^{FAD+Mn+D\text{-}LAC}$ structure is colored in orange.

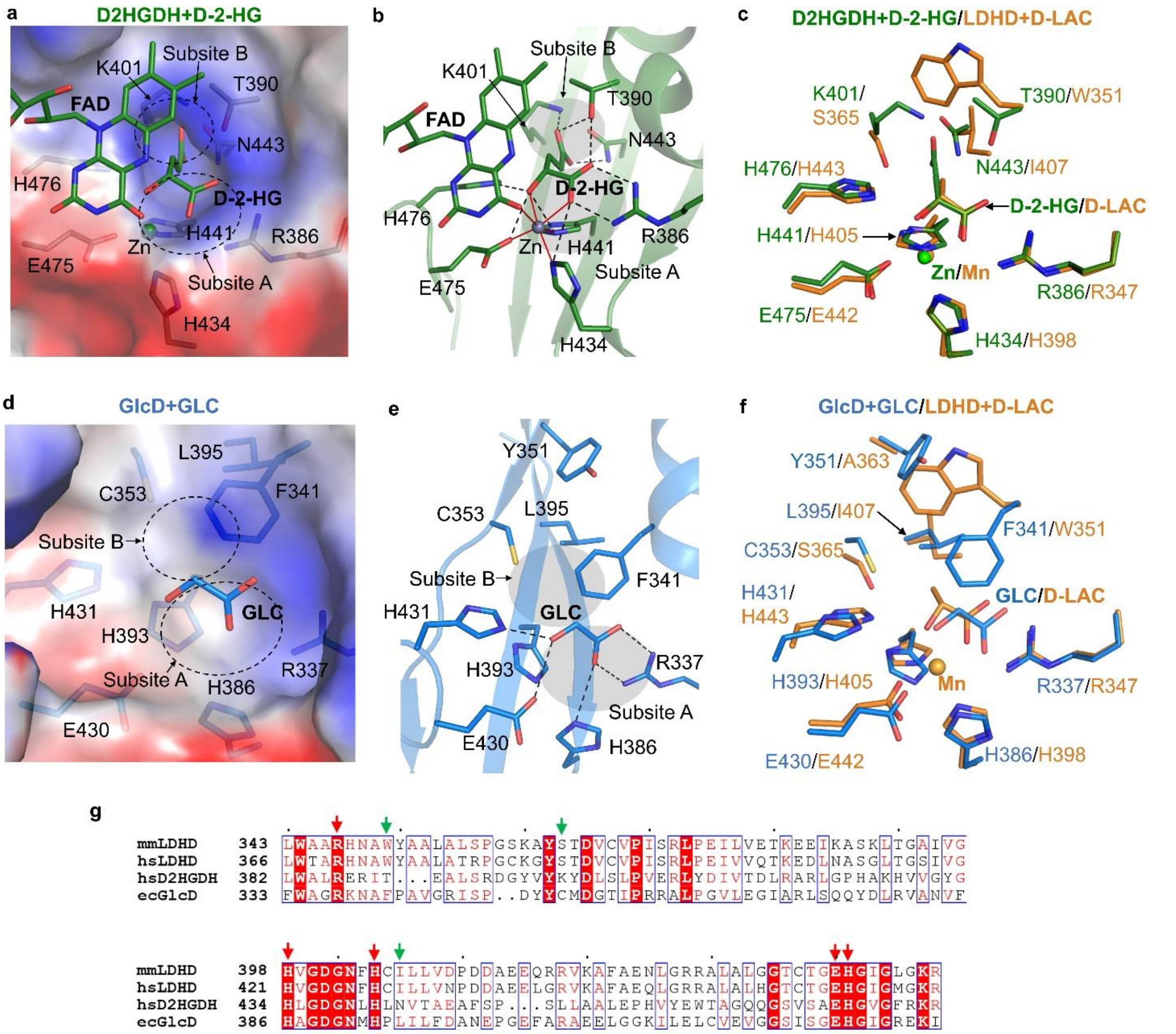

Supplementary Fig 12. Comparison of the substrate-binding sites of LDHD, D2HGDH and GlcD. a. Electrostatic surface of the substrate-binding site of human D2HGDH (hsD2HGDH) bound with D-2-HG and Zn^{2+} (PDB 6LPP). **b.** Interactions between D-2-HG, Zn^{2+} and the surrounding residues. The hydrogen bonds are shown in black dashed lines and the coordinate bonds are shown in red solid lines. **c.** Superposition of the key residues composing the substrate-binding sites of mouse LDHD and human D2HGDH bound with D-LAC and D-2-HG, respectively. **d.** Electrostatic surface of the substrate-binding site of the predicted model of *E. coli* GlcD (ecGlcD) bound with substrate glycolate (GLC). **e.** Interactions between GLC and the surrounding residues. The hydrogen bonds are shown in black dashed lines. **f.** Superposition of the key residues composing the substrate-binding sites of mouse LDHD and *E. coli* GlcD with bound substrates D-LAC and GLC, respectively. **g.** Sequence alignment of mmLDHD, hsLDHD, hsD2HGDH, and ecGlcD showing the key residues forming the substrate-binding site. Residues located at the substrate-binding subsite A are indicated by red arrows, and those at the substrate-binding subsite B are indicated by green arrows.

Supplementary Table 1. Functional roles of LDHD mutations in the catalytic reaction and pathogenesis.

mLDHD	hLDHD	Specific activity ($\mu m \cdot min^{-1} \cdot mg^{-1}$) [a]	Residual activity compared to WT	Structure location	Functional role
WT	-	1.083 ± 0.003	-	-	-
Active-site mutations					
R347A	R370A	N.D. [b]	N.D.	α9	Impair substrate binding
H398A	H421A	N.D.	N.D.	β13-η5 loop	Impair metal ion binding
H405A	H428A	N.D.	N.D.	β14	Impair metal ion binding
E442A	E465A	N.D.	N.D.	α11-α12 loop	Impair metal ion binding
H443A	H466A	N.D.	N.D.	α11-α12 loop	Impair substrate binding
Disease-associated mutations					
T228M	T251M	0.024 ± 0.02	2.2%	η4-β7 loop	Impair FAD binding
R347W	R370W	0.045 ± 0.027	4.2%	α9	Impair substrate binding
W351C	W374C	0.127 ± 0.014	11.8%	α9	Impair substrate binding
T440M	T463M	0.014 ± 0.005	1.3%	α11-α12 loop	Impair FAD binding

[a] The specific activities of WT and mutant mLDHD enzymes were measured at the standard conditions: 50 mM Tris-HCl (pH 7.4), 2 μg enzyme, 50 μM $MnCl_2$, 200 μM PMS, 100 μM DCIP, and 1 mM D-lactate. The molar ratio of FAD:protein was determined to be 0.73 ± 0.04 for WT mLDHD, 0.44 ± 0.02 for the W351C mutant, and 0.48 ± 0.008 for the R347W mutant, respectively. The specific activities of WT and mutant mLDHD enzymes were corrected according to the concentration of active enzyme in the reaction solution calculated based on the FAD occupancy. The specific activity values are presented as the mean ± SEM (n = 3 independent experiments).
[b] N.D., not detectable.
