# Supplementary material for: Lactate dehydrogenase D is a general dehydrogenase for D-2-hydroxyacids and is associated with D-lactic acidosis
Source: Nat Commun. 2023 Oct 20;14:6638. doi: 10.1038/s41467-023-42456-3 (PMC10589216; doi:10.1038/s41467-023-42456-3)
Supplement: Supplementary file 3 — Reporting Summary [file 41467_2023_42456_MOESM3_ESM.pdf]

nature portfolio

Corresponding author(s): Jianping Ding

Last updated by author(s): Oct 8, 2023

Reporting Summary

Nature Portfolio wishes to improve the reproducibility of the work that we publish. This form provides structure for consistency and transparency in reporting. For further information on Nature Portfolio policies, see our Editorial Policies and the Editorial Policy Checklist.

Statistics

For all statistical analyses, confirm that the following items are present in the figure legend, table legend, main text, or Methods section.

n/a	Confirmed	
☐	☒	The exact sample size (*n*) for each experimental group/condition, given as a discrete number and unit of measurement
☐	☒	A statement on whether measurements were taken from distinct samples or whether the same sample was measured repeatedly
☐	☒	The statistical test(s) used AND whether they are one- or two-sided<br>*Only common tests should be described solely by name; describe more complex techniques in the Methods section.*
☒	☐	A description of all covariates tested
☐	☒	A description of any assumptions or corrections, such as tests of normality and adjustment for multiple comparisons
☐	☒	A full description of the statistical parameters including central tendency (e.g. means) or other basic estimates (e.g. regression coefficient) AND variation (e.g. standard deviation) or associated estimates of uncertainty (e.g. confidence intervals)
☐	☒	For null hypothesis testing, the test statistic (e.g. *F*, *t*, *r*) with confidence intervals, effect sizes, degrees of freedom and *P* value noted<br>*Give P values as exact values whenever suitable.*
☒	☐	For Bayesian analysis, information on the choice of priors and Markov chain Monte Carlo settings
☒	☐	For hierarchical and complex designs, identification of the appropriate level for tests and full reporting of outcomes
☒	☐	Estimates of effect sizes (e.g. Cohen's *d*, Pearson's *r*), indicating how they were calculated

Our web collection on statistics for biologists contains articles on many of the points above.

Software and code

Policy information about availability of computer code

Data collection	X-ray diffraction data were collected at BL02U1 of Shanghai Synchrotron Radiation Facility (SSRF) and BL18U1 and BL19U1 of National Facility for Protein Science in Shanghai (NFPSS). Enzymatic activity data was collected using Gen5 software (v2.09) integrated with Synergy Neo2 microplate reader. Size exclusion chromatography data was collected using Unicorn (v7.0) integrated with AKTA chromatography system.
Data analysis	HKL2000 (v718), XDS or autoPROC was used to process X-ray diffraction data. Phenix (v1.18.2) was used to perform molecular replacement and structure refinement. Coot (v0.8.2) was used to perform model building. Pymol (v2.4.1) was used to perform structural analysis and generate structure figures. Yasara (v17.8.15) was used to build structural models of mutants. PR.ThermControl (v2.3.1) was used to perform protein thermostability analysis. GraphPad Prism (v7.0) was used to generate the figures for the enzymatic activity assays.

For manuscripts utilizing custom algorithms or software that are central to the research but not yet described in published literature, software must be made available to editors and reviewers. We strongly encourage code deposition in a community repository (e.g. GitHub). See the Nature Portfolio guidelines for submitting code & software for further information.

Data

Policy information about availability of data

All manuscripts must include a data availability statement. This statement should provide the following information, where applicable:

- Accession codes, unique identifiers, or web links for publicly available datasets
- A description of any restrictions on data availability
- For clinical datasets or third party data, please ensure that the statement adheres to our policy

Atomic coordinates and structure factors of the following structures have been deposited with the Protein Data Bank (PDB): mLDHD in complex with FAD (PDB 8JDC [https://doi.org/10.2210/pdb8JDC/pdb]), mLDHDH405A in complex with FAD (PDB 8JDD [https://doi.org/10.2210/pdb8JDD/pdb]), mLDHD in complex with FAD, Mn2+ and D-lactate (PDB 8JDE [https://doi.org/10.2210/pdb8JDE/pdb]), mLDHDH405A in complex with FAD and D-lactate (PDB 8JDF [https://doi.org/10.2210/pdb8JDF/pdb]), mLDHDH405A in complex with FAD and D-2-hydroxybutyrate (PDB 8JDG [https://doi.org/10.2210/pdb8JDG/pdb]), mLDHDH405A in complex with FAD and D-2-hydroxyvalerate (PDB 8JDN [https://doi.org/10.2210/pdb8JDN/pdb]), mLDHDH405A in complex with FAD and D-2-hydroxyhexanoate (PDB 8JDO [https://doi.org/10.2210/pdb8JDO/pdb]), mLDHDH405A in complex with FAD and D-2-hydroxyoctanoate (PDB 8JDB; https://doi.org/10.2210/pdb8JDB/pdb]), mLDHDH405A in complex with FAD and D-2-hydroxyisovalerate (PDB 8JDP; https://doi.org/10.2210/pdb8JDP/pdb]), mLDHDH405A in complex with FAD and D-2-hydroxyisocaproate (PDB 8JDQ [https://doi.org/10.2210/pdb8JDQ/pdb]), mLDHDH405A in complex with FAD and D-2-hydroxy-3-methylvalerate (PDB 8JDR [https://doi.org/10.2210/pdb8JDR/pdb]), mLDHD in complex with FAD, Mn2+ and pyruvate (PDB 8JDS [https://doi.org/10.2210/pdb8JDS/pdb]), mLDHD in complex with FAD, Mn2+ and 2-ketobutyrate (PDB 8JDT [https://doi.org/10.2210/pdb8JDT/pdb]), mLDHD in complex with FAD, Mn2+ and 2-ketovalerate (PDB 8JDU [https://doi.org/10.2210/pdb8JDU/pdb]), mLDHD in complex with FAD, Mn2+ and 2-ketohexanoate (PDB 8JDV [https://doi.org/10.2210/pdb8JDV/pdb]), mLDHD in complex with FAD, Mn2+ and 2-ketoisovalerate (PDB 8JDX [https://doi.org/10.2210/pdb8JDX/pdb]), mLDHD in complex with FAD, Mn2+ and 2-ketoisocaproate (PDB 8JDY [https://doi.org/10.2210/pdb8JDY/pdb]), and mLDHD in complex with FAD, Mn2+ and 2-keto-3-methylvalerate (PDB 8JDZ [https://doi.org/10.2210/pdb8JDZ/pdb]).

Research involving human participants, their data, or biological material

Policy information about studies with human participants or human data. See also policy information about sex, gender (identity/presentation), and sexual orientation and race, ethnicity and racism.

Reporting on sex and gender	Sex and gender information is not relevant to this study.
Reporting on race, ethnicity, or other socially relevant groupings	Race, ethnicity, or other socially relevant grouping information is not relevant to this study.
Population characteristics	This study did not involve population characteristics.
Recruitment	This study did not involve recruitment of participants.
Ethics oversight	This study did not involve any ethics oversight organizations.

Note that full information on the approval of the study protocol must also be provided in the manuscript.

Field-specific reporting

Please select the one below that is the best fit for your research. If you are not sure, read the appropriate sections before making your selection.

☒ Life sciences ☐ Behavioural & social sciences ☐ Ecological, evolutionary & environmental sciences

For a reference copy of the document with all sections, see nature.com/documents/nr-reporting-summary-flat.pdf

Life sciences study design

All studies must disclose on these points even when the disclosure is negative.

Sample size	Sample size was not predetermined statistically. X-ray diffraction data were collected on single crystals until acceptable completeness and redundancy were reached. This sample size was considered sufficient as it provided enough signal for structure determination, and all statistics of diffraction data and structure refinement fell within acceptable ranges.<br>For biochemical study, the WT and mutant proteins were assayed for different substrates in three independent experiments.
Data exclusions	No data were excluded from the analysis.
Replication	The biochemical experiments were repeated three times independently. All attempts at replication were successful.
Randomization	5% of reflections for each dataset were randomly allocated by Phenix program for R-free calculation.
Blinding	The study is based on crystallographic and in vitro biochemical experiments, devoid of any human individuals, animals or cells. These experiments require precise addition of distinct compounds into the crystallization drops or the reaction solution by the investigator. Therefore, blinding is not applicable in this study.

Reporting for specific materials, systems and methods

We require information from authors about some types of materials, experimental systems and methods used in many studies. Here, indicate whether each material, system or method listed is relevant to your study. If you are not sure if a list item applies to your research, read the appropriate section before selecting a response.

Materials & experimental systems

n/a	Involved in the study
☒	☐ Antibodies
☒	☐ Eukaryotic cell lines
☒	☐ Palaeontology and archaeology
☒	☐ Animals and other organisms
☒	☐ Clinical data
☒	☐ Dual use research of concern
☒	☐ Plants

Methods

n/a	Involved in the study
☒	☐ ChIP-seq
☒	☐ Flow cytometry
☒	☐ MRI-based neuroimaging
