# Supplementary material for: Lactate dehydrogenase D is a general dehydrogenase for D-2-hydroxyacids and is associated with D-lactic acidosis
Source: Nat Commun. 2023 Oct 20;14:6638. doi: 10.1038/s41467-023-42456-3 (PMC10589216; doi:10.1038/s41467-023-42456-3)
Supplement: Supplementary file 4 — Description of Additional Supplementary Files [file 41467_2023_42456_MOESM4_ESM.pdf]

Description of Additional Supplementary Files

File Name: Supplementary Data 1

Description: Statistics of diffraction data and structure refinement

Footnotes:

[a] Numbers in parentheses represent the highest resolution shell.

[b] $R_{merge} = \sum_{hkl}\sum_{i}|I_i (hkl) - <I (hkl)>|/\sum_{hkl}\sum_{i}I_i (hkl)$.

[c] $R = \sum_{hkl}||Fo|-|Fc||/\sum_{hkl}|Fo|$.
